# Decolonizing global health—what does it mean for us?

**DOI:** 10.1093/eurpub/ckad001

**Published:** 2023-06-01

**Authors:** Birger C Forsberg, Jesper Sundewall

**Affiliations:** Department of Global Public Health, Karolinska Institutet, Stockholm, Sweden; Social Medicine and Global Health, Lund University, Lund, Sweden; HEARD, University of KwaZulu-Natal, Durban, South Africa

The calls for decolonizing global health are increasing in both numbers and intensity. The discourse is undoubtedly gaining traction and increasingly a topic for discussion in journals, the public global health debate and at international conferences. A natural question that begs to be answered in the European public health community is ‘What does it mean for us?’ Here, we provide a few possible answers to that question.

The concept of decolonizing global health stems from the persisting power inequities in relationships between global health actors and researchers in what is bluntly referred to as the global ‘North’ (high-income countries) and the ‘South’ [low- and lower middle-income countries; LMICs^1^ (The use of the categories low- and middle-income countries has recently been challenged in Lencucha and Neupane.^2^)] This in turn is a reflection that much of global health cooperation and research is funded by development assistance and the structures around how that aid is provided. Cooperations were originally formed in the era of colonialism and under colonial relations. Scholars in the decolonization debate have proposed that cooperation between ‘donors’ and ‘recipients’ today is still largely influenced by such colonial relations.^3^

Others have suggested that the current global health ideology should be understood in a wider context in which that ideology contributes to preserving an unjust world order and related inequalities. They claim that the wealth and power distribution are not put at stake but rather reinforced by the ideology.^4^ This refers to both control of funds and the claim that persons in, or from, the ‘South’ are inadequately respected in the discipline, and that they are not properly represented in decision-making.^5^

The call to ‘decolonize global health’ certainly has many valid points that require our attention as a public health community in Europe, a part of the ‘North’. We argue that action must be taken at both the institutional and individual levels.

It seems of priority that a common platform is created for establishing an evidence base to document how global health research funds flow from funders to the ultimate target groups. For instance, mapping how these funds are distributed and absorbed at different levels (‘North’ vs. ‘South’; globally, nationally, regionally, locally; etc.) would be valuable. The distribution of power will be detrimental if it leads to unjust use of resources, leading to increasing inequities. There is need for more research on the actual use and efficiency of international funds for global health research and implementation. Funders have different strategies and facts could provide important lessons on how well their funds achieve intended objectives in the ‘South’, if at all.

The movement to decolonize global health provides an impetus, but also opportunities, for European institutions to reorient themselves. Universities can give more priority to students from LMICs in masters and doctoral training programs. They can also work more actively with twinning their research programs and funds with institutions in LMIC, allowing both research outputs and degrees to occur as often at partner universities as at European universities. This will challenge research foundations to find new ways to merit twinning applications and disburse funds fairly. European researchers and institutions must be prepared to make major contributions to the growth of research capacities in LMIC. To remain relevant in global health, European universities should to a larger extent reflect the world at large and recognize the rapidly growing capacity and competence of global health researchers in the South.

At the individual level, global health researchers at institutions in the ‘North’ should strive to include researchers from the ‘South’ in all steps of relevant research, ensuring joint and equal ownership over research data, and from the onset have a plan for fair distribution of authorship over publications.

Calls for decolonization should perhaps be better understood as calls for more equitable partnerships and a better power balance between the ‘South’ and the ‘North’. The European public health research community must take on this challenge. It is time for serious introspection and a discussion about how we can transform in ways that truly share power and resources for a more equitable world.

## Data Availability

This viewpoint paper does not provide any new data, nor is it based on a specific data set.

## References

[1] World Bank Country and Lending Groups. *World Bank*. Washington: https://datahelpdesk.worldbank.org/knowledgebase/articles/906519-world-bank-country-and-lending-groups%20at%20November%2011 (accessed 22 November 2022).

[2] Lencucha R , Neupane S. The use, misuse and overuse of the ‘low-income and middle-income countries’ category. BMJ Glob Health 2022;7:e009067. 10.1136/bmjgh-2022-009067 PMC9185671 35672116

[3] Khan M , Abimbola S, Aloudat T, et al Decolonising global health in 2021: A roadmap to move from rhetoric to reform. BMJ Glob Health 2021;6:e005604. 10.1136/bmjgh-2021-005604 PMC7993212 33758016

[4] Kim H. The implicit ideological function of the global health field and its role in maintaining relations of power. BMJ Glob Health 2021;6:e005620. 10.1136/bmjgh-2021-005620 PMC8054087 33853846

[5] Hellowell M , Nayna Schwerdtle P. Powerful ideas? Decolonisation and the future of global health. BMJ Glob Health 2022;7:e006924. 10.1136/bmjgh-2021-006924 PMC8785167 35064046

